# Vitamin C Modes of Action in Calcium-Involved Signaling in the Brain

**DOI:** 10.3390/antiox12020231

**Published:** 2023-01-19

**Authors:** Ludmila Zylinska, Malwina Lisek, Feng Guo, Tomasz Boczek

**Affiliations:** 1 Department of Molecular Neurochemistry, Medical University of Lodz, 92215 Lodz, Poland; 2 Department of Pharmaceutical Toxicology, China Medical University, Shenyang 110122, China

**Keywords:** vitamin C, calcium, brain, neurotransmission, regulation, calcium channels, nitric oxide

## Abstract

Vitamin C (ascorbic acid) is well known for its potent antioxidant properties, as it can neutralize ROS and free radicals, thereby protecting cellular elements from oxidative stress. It predominantly exists as an ascorbate anion and after oxidation to dehydroascorbic acid and further breakdown, is removed from the cells. In nervous tissue, a progressive decrease in vitamin C level or its prolonged deficiency have been associated with an increased risk of disturbances in neurotransmission, leading to dysregulation in brain function. Therefore, understanding the regulatory function of vitamin C in antioxidant defence and identification of its molecular targets deserves more attention. One of the key signalling ions is calcium and a transient rise in its concentration is crucial for all neuronal processes. Extracellular Ca^2+^ influx (through specific ion channels) or Ca^2+^ release from intracellular stores (endoplasmic reticulum, mitochondria) are precisely controlled. Ca^2+^ regulates the functioning of the CNS, including growth, development, myelin formation, synthesis of catecholamines, modulation of neurotransmission and antioxidant protection. A growing body of evidence indicates a unique role for vitamin C in these processes. In this short review, we focus on vitamin C in the regulation of calcium-involved pathways under physiological and stress conditions in the brain.

## 1. Introduction

Although ascorbic acid was discovered and named by Casimir Funk as “vitamin C” 110 years ago [1], and more than 70,000 research papers in PubMed have been published until now, many aspects of vitamin C’s function still remain a mystery. Vitamin C is well known mainly for its prevailing antioxidant properties and the ability to neutralize a wide range of reactive oxygen (ROS) and nitrogen (RNS) species [2,3]. Thereby, it can protect cellular macromolecules—proteins, lipids, carbohydrates and nucleic acids—from oxidative damage [4]. ROS and RNS are physiologically produced during many metabolic reactions in mitochondria, endoplasmic reticulum, lysosomes, peroxisomes, cytosol and plasma membrane [5,6]. The imbalance between oxidant level and the capacity of defence the system can lead to pathological conditions, including atherosclerosis, cardiovascular disease, chronic inflammatory diseases or cancer [7,8]. Particularly, ROS/RNS concentration increases significantly in neurodegenerative disorders, depression or cognitive impairment [9,10]. Similar changes caused by oxidative and nitrosative stress have also been observed during aging [11,12]. Another vitamin C function is linked with its activity as a co-factor in the synthesis of certain amino acids, carnitine, catecholamines, collagen, cholesterol and a few peptide hormones. Moreover, vitamin C has been shown to regulate the function of nitric oxide synthase (NOS) by recycling its cofactor—tetrahydrobiopterin (BH_4_) [13]. Recently, vitamin C has been established as an epigenetic regulator during neurodevelopment and may play a direct role in the expression of hundreds of different genes [14,15].

Calcium ions are ubiquitous intracellular messengers that coordinate processes crucial for brain physiology and play an important role in synaptic transmission and synaptic plasticity. Intracellular Ca^2+^ increase can be reached by the influx from extracellular space through two types of plasma membrane Ca^2+^ channels: voltage-dependent Ca^2+^ channels (VDCCs) with subtypes N, L, P/Q, R, T and ligand-gated ionotropic channels, which include α-amino-3-hydroxy-5-methyl-4-isoxazolepropionic acid receptors (AMPARs), N-methyl-D-aspartate receptors (NMDARs) and kainate receptors, all activated by glutamate [16,17].

In contrast to the ionotropic receptors, metabotropic receptors are not ion channels but regulate calcium homeostasis by mobilizing calcium from internal stores via GTP binding protein-dependent mechanisms [18]. Ca^2+^ can be released from the endoplasmic reticulum (ER) by inositol-1,4,5-triphosphate receptors (IP_3_Rs) and ryanodine receptors (RyRs) upon activation of some phospholipase C (PLC)-linked G protein-coupled receptors (GPCRs) [19,20,21,22]. Restoration of cytosolic Ca^2+^ concentration is facilitated by the sarco (endo)plasmic reticulum Ca^2+^-ATPase (SERCA), secretory pathway Ca^2+^-ATPase (SPCA) or mitochondrial uniporter (MCU), which operate in the ER, Golgi apparatus and mitochondria, respectively [23,24,25,26]. In addition, Ca^2+^ is effectively removed to the extracellular environment by the plasma membrane Ca^2+^-ATPase (PMCA) and Na^+^/Ca^2+^exchanger (NCX) [27,28,29,30]. On the other hand, a number of cytosolic calcium-binding proteins have been characterized as buffering proteins, also able to control intracellular calcium concentration [31].

Disruption in neuronal calcium homeostasis can occur after excessive release of glutamate from the neurons or glia, with subsequent activation of NMDARs and enhanced Ca^2+^ influx, which may generate the overproduction of ROS/RNS. Massive Ca^2+^ accumulation triggers mitochondrial damage activating mitochondrial membrane permeability transition (MPT) and production of reactive species, leading to cell death [32]. In this short review, we summarize the recent knowledge about cellular and molecular mechanisms concerning vitamin C action and regulation of Ca^2+^ signalling in the brain under physiological and stress conditions.

## 2. Vitamin C Turnover in Humans

In contrast to many mammals, vitamin C cannot be synthesized in humans due to the absence of a rate-limiting enzyme—gulonolactone oxidase—that is encoded by the *GULO* gene [33]. Thus, humans must be supplemented with vitamin C from external sources. At physiological pH, it is present in the anionic form (ascorbate) and could be oxidized to an unstable form—dehydroascorbic acid (DHA)—which can be further reduced to ascorbate at the expense of NADPH or glutathione [34]. Transport of ascorbate from the distal ileum to the blood is mediated by the ubiquitous sodium-dependent vitamin C transporter 1 (SVCT1) [35]. Next, it is actively transported to the cerebrospinal fluid (CSF) by the epithelial cells of the choroid plexus and further to the brain by sodium-dependent vitamin C transporter 2 (SVCT2), which is the only transporter expressed in the brain, predominantly in neurons [36,37]. It allows for vitamin C accumulation against the concentration gradient. In neuron-rich areas, its concentration may achieve the value up to 10 mM, in contrast to 40–60 μM detected in plasma [37]. Relatively less DHA is transported to neurons, as this process engages the glucose transporters (GLUTs) possessing significantly higher affinity for glucose [38]. Contrarily, DHA concentration in astrocytes is significantly higher because it enters the cell mainly through GLUT1. The average half-life of vitamin C in an adult human is about 10–20 days and vitamin C and its metabolites (dehydroascorbic acid, 2,3-diketogulonic acid and oxalic acid) are next eliminated through urine [39].

## 3. Vitamin C and Neurotransmission

In the brain, vitamin C has been shown to act as a neuromodulator of glutamatergic, dopaminergic, adrenergic, cholinergic and GABAergic signalling; however, different mechanisms of regulation have been identified in distinct types of neurons. To be able to carry the message from cell to cell, neurotransmitters must be synthesized, packed, secreted and, finally, inactivated. Vitamin C has been shown to affect at least two stages of neurotransmitter action: synthesis and release in the brain. One of the most prominent processes engaging vitamin C is the recycling of tetrahydrobiopterin (BH_4_), a co-factor for several enzymes responsible for the synthesis of neurotransmitters. For example, serotonin is produced from 5-HTP, and 5-HTP is made from L-tryptophan by tryptophan hydroxylase, the enzyme that requires BH_4_ [40]. BH_4_ is necessary for tyrosine hydroxylase activity during synthesis of L-DOPA, as well as in the next step, when noradrenaline is generated from dopamine in secretory granules [41,42]. In this reaction, vitamin C acts as a co-factor for dopamine β hydroxylase. In cultured SH-SY5Y neuroblastoma cells, the synthesis of noradrenaline was stimulated several-fold by intracellular ascorbate. Interestingly, treatment with 100 μM dopamine greatly increased cellular superoxide generation, which was prevented by ascorbate loading, additionally showing a protective role of vitamin C [41].

Another example of its modulatory action can be the regulation of catecholamine and acetylcholine release from synaptic vesicles [37,43]. Early studies demonstrated that vitamin C modulated vesicular acetylcholine release from rat brain synaptosomes [44] and cultured adrenal chromaffin cells [45]. Moreover, since vitamin C is present at high concentrations in synaptic vesicles of glutamatergic neurons, it could be co-released along with the classical neurotransmitters—glutamic acid and noradrenaline [46]. On the other hand, it has been well documented that neurotransmitters can regulate the release and uptake of vitamin C and that bi-directional communication between these processes allows for fine-tuning of the homeostatic response to environmental stimuli. Several studies have documented that SVCT2 can be regulated by multiple signalling pathways, including phosphorylation by protein kinases PKA and PKC, as well as by activation of the NO/cGMP/PKG/NF-kB pathway [47,48,49]. The release of neurotransmitters in the CNS is mediated by exocytosis, but they are also liberated by the reversion of vitamin C transporters. In primary retinal cultures, dopamine controlled the release of ascorbate from retinal neurons by the reversal of the SVCT2, involving D1receptor/cAMP/EPAC2 pathway, and this was a dose-dependent effect [50]. Additionally, dopamine-dependent SVCT2 reversion leading to ascorbate release can also occur indirectly by activation of AMPA/kainate receptors and downstream ERK/AKT pathways [51]. In this case, dopamine can induce a small release of glutamate, which next activates AMPA/kainate receptors, increasing vitamin C release. It has been previously shown that in cultured avian retinal cells, glutamate, by activating its ionotropic receptors, caused ascorbate release via the reversion of SVCT2 [52]. Taking into account that the dopaminergic and glutamatergic systems are interconnected, both neurotransmitters can mutually contribute to ascorbate release, but distinct downstream pathways can be engaged.

## 4. Vitamin C and Neuronal Calcium Signalling

It is generally well accepted that neurotransmitter release is a Ca^2+^-dependent event. However, recent studies suggest that local and transient, but not global, changes in discrete microdomains of the plasma membrane are much more efficient triggers of exocytosis [53,54]. Moreover, a growing body of evidence demonstrates the existence of a physical and functional connection between the ER and mitochondria via the junctions called mitochondria–endoplasmic contacts (MERCs) that can control the release of neurotransmitters [55,56].

Neurotransmitters act in the brain through membrane-localized specific receptors that can be categorized into two classes. The first one includes extracellular ligand-gated ion channel receptors (ionotropic receptors), which allow selected ions to pass through and are responsible for fast transmission. The second one comprises GPCRs (metabotropic receptors), which, after the binding of neurotransmitters, alter their association with heterotrimeric G proteins, thereby indirectly activating signalling through a variety of intracellular pathways, including those generating second messengers. This can provoke changes in intracellular cAMP, Ca^2+^, diacylglycerol (DAG) or inositol 1,4,5-triphosphate (IP_3_) concentrations and trigger the activation of diverse downstream signalling cascades.

Under physiological conditions, vitamin C is assumed to regulate neurotransmission mainly by limiting excitability that results from a rapid and transient rise in the intracellular calcium level. Many reports indicate that this protective effect is associated with vitamin C redox potential [57]. There are also some studies showing more versatile types of vitamin C action. Antioxidant function of ascorbate is mainly linked with the scavenging of ROS and RNS. However, ascorbate can also function as a pro-oxidant by catalysing the formation of several ROS. This may occur during the reduction in transition metals via a one-electron transfer mechanism or oxygen via a two-electron transfer mechanism. Metal-catalysed oxidation (MCO) has been shown to modify proteins, often resulting in alterations in their structure and loss of activity and/or function [58].

### 4.1. Vitamin C in the Regulation of Calcium Channels

**Figure 1:**
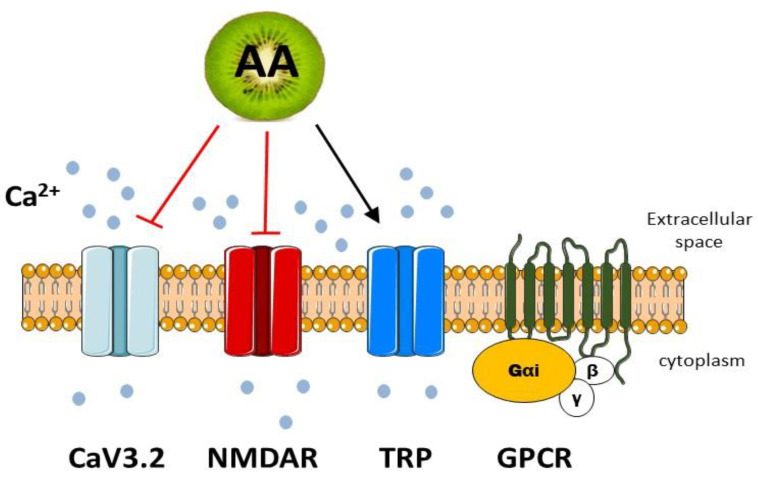
The regulation of Ca^2+^-permeable channels by vitamin C. CaV3.2—T-type voltage-dependent Ca^2+^ channel; NMDAR—N-methyl-D-aspartate receptor; TRP—transient receptor potential channel; GPCR—G-protein-coupled receptor; AA—ascorbic acid (vitamin C). Red arrow—inhibition; black arrow—activation.

There are several groups of calcium channels responsible for the influx of Ca^2+^ into the cells. They differ in structure, localization and functional properties [59] and regulation by vitamin C (Figure 1). The four major channel categories include voltage-gated calcium channels (VGCCs), receptor-operated calcium channels (ROCCs), store-operated channels (SOCs) and transient receptor potential (TRP) channels [16,60,61].

VGCCs comprise high voltage-activated (L- and P/Q-types), intermediate voltage-activated (R-type) and low voltage-activated (T-type) that are important mediators of depolarization-evoked release of neurotransmitters.

Receptor operated calcium channels (ROCCs) are metabotropic cation channels that, following stimulation of GPCRs, initiate a cascade of pathways via G proteins and contribute to phospholipase C-dependent Ca^2+^ entry. They can also act through receptor tyrosine kinases.

Transient receptor potential (TRP) channels are another group of calcium channels, which are localized in the plasma membrane and in the membrane(s) of intracellular organelles in nearly all living cells. They have been classified into six subfamilies, including TRPC (*canonical*), TRPV (*vanilloid*), TRPM (*melastatin*), TRPA (*ankyrin*), TRPML (*mucolipin*) and TRPP (*polycystic*).

#### 4.1.1. Vitamin C in the Regulation of VGCC

Up to now, low-voltage-activated T-type Ca^2+^ channels have been identified as a direct target for vitamin C action. Subtypes of T-type channels include Cav3.1, Cav3.2 and Cav3.3 that are mostly distributed at cell bodies and dendrites of neurons in various brain areas (olfactory bulb, amygdala, cerebellar cortex, hippocampus, thalamus, hypothalamus, striatum) [62,63]. These channels can regulate glutamate neurotransmission in peripheral nerve endings of nociceptors, and Ca_V_3.2 T-type channels have a pivotal role in the processing of pain signals [64,65].

Molecular studies revealed that ascorbate inhibits Cav3.2 currents by initiating the metal-catalysed oxidation of histidine residue (H^191^) that is recognized as a part of the high-affinity metal-binding site located on the external surface of Cav3.2 [66]. Vitamin C selectively suppresses Ca_V_3.2 T-type channels in dorsal root ganglion cells and thalamic slices via simultaneous binding of Zn^2+^ and vitamin C by His^191^ residue [67]. Interestingly, the same histidine residue was shown to be involved in a mechanism of Cav3.2 current blockage in response to nitrous oxide. Inhibitory potency of ascorbate has also been observed for recombinant human and rat Cav3.2 channels heterologously expressed in HEK 293 cells. Moreover, concentrations of vitamin C that inhibited T currents were within its physiological concentrations, and neither Cav3.1 nor Cav3.3 was altered under these conditions.

Ascorbic acid was also shown to inhibit Ca_v_3.2 channels in NG108-15 cells. In cells treated with H_2_S, a gasotransmitter that induces neuronal differentiation via activation of T-type Ca^2+^ channels, the presence of ascorbate abolished the neuritogenic effect [68]. The series of patch clamp experiments performed with those cells showed that T currents facilitated by NaHS were reduced by ascorbic acid, indicating the involvement of Ca_v_3.2 [69].

There is increasing evidence that T-type calcium channels are involved in the processing of pain signals. In particular, Ca_v_3.2 isoform, which is highly expressed in the peripheral ending of nociceptors, seems to contribute to the pathophysiology of neuropathic pain [70,71,72,73]. T-type calcium channels do not play a role in the processing of nociceptive signals under physiological conditions but contribute to the persistent hyperalgesia in pathological states.

#### 4.1.2. Vitamin C in the Regulation of NMDARs

One of the most active groups of receptors that transmits glutamate signalling in the brain is the family of ionotropic NMDA receptors (NMDARs). They form cation-permeable channels that mainly transport Ca^2+^ but also Na^+^ and K^+^. Calcium entry through these channels regulates many neuronal functions, including neurodevelopment, synaptic activity, release of neurotransmitters as well as neuroprotection [74,75]. NMDAR forms tetraheteromeric structures that differ in subunit composition and are classified as GluN1, GluN2A, GluN2B, GluN2C, GluN2D, GluN3A and GluN3B [76]. The composition of synaptic NMDARs is crucial for a proper neuronal functioning and regulation of Ca^2+^-dependent downstream signalling cascades associated with receptor activation. One of the interesting regulatory networks is created via interaction of Ca^2+^ with calmodulin (CaM). CaM, a ubiquitous calcium sensor protein with a very high Ca^2+^ affinity, is present in the brain in the highest concentration and can regulate nearly 300 proteins [77]. The Ca^2+^/CaM complex activates many enzymes, including nNOS, adenylate and guanylate cyclases, cyclic nucleotide phosphodiesterase, calcineurin, Ca^2+^/CaM-dependent protein kinases, PMCA, certain transcription factors and many others [78,79,80,81,82,83,84]. Thus, the participation of vitamin C in a direct and/or indirect control of Ca^2+^ signals may have profound consequences for brain function, as well as for vulnerability to injury and diseases.

Vitamin C has the ability to modulate glutamatergic neurotransmission because the distribution of glutamatergic NMDARs is the highest in those brain areas where vitamin C concentration is also high, i.e., in the cortex, amygdala and hippocampus [85,86]. An early study demonstrated that vitamin C inhibited glutamate binding to NMDARs and decreased NMDA currents in isolated cortical neurons [87]. Further research on cultured rat cerebral cortical neurons revealed the full protection from toxicity induced by NMDA and glutamate, as well as significantly diminishing cell death [88]. The effects of ascorbate could be due to redox modulation of NMDAR or direct scavenging of ROS generated by receptor activation.

Another interesting observation was made on cultured retinal cells and intact retinal tissue, where ascorbate and dehydroascorbate promoted an accumulation of extracellular glutamate by inhibition of glutamate uptake [89]. In addition, ascorbate decreased the level of excitatory amino acid transporter 3 (EAAT3). Vitamin C also enhanced NMDAR activity and increased the internalization of GluN1 NMDAR subunits. Both compounds increased the phosphorylation level of cAMP response element-binding protein (CREB) in neuronal nucleus in a glutamate receptor and Ca^2+^/CaM kinase-dependent manner. CREB is a critical nuclear transcription factor involved in proliferation, differentiation, survival, long-term synaptic potentiation, neurogenesis and neuronal plasticity.

It should be mentioned that the effects exerted by vitamin C also depend on the mechanism of its transport. For example, the cultured chick retinal cells were able to take up ascorbate in a time- and dose-dependent manner, and next, activation of ionotropic glutamate receptors induced the release of vitamin C in response to glutamate, NMDA or kainate in a Na^+^-dependent but Ca^2+^-independent manner [52]. The authors suggested that the activation of NMDARs promoted glutamate release, which, in turn, activated non-NMDARs and induced the release of vitamin C. Moreover, inhibition of the uptake and release by removal of sodium ions indicated the involvement of SVCT-2, suggesting the presence of a high-affinity and bidirectional transport system for ascorbate. The findings from another study showed an absolute SVCT2 requirement for Ca^2+^ and Mg^2+^ [90]. The transport cycle can be regulated by both cations by switching the transporter from an inactive to an active conformation by increasing Vmax without affecting Km of the transporter or its cooperativity with Na^+^. It has been concluded that SVCT2 acting as a Ca^2+^/Mg^2+^-dependent co-transporter of Na^+^ and vitamin C can cooperate with glutamate-triggered neurotransmission in a cell-type-specific mode.

The release of ascorbate from glutamatergic neurons is a part of the glutamate reuptake process as glutamate transporters can exchange ascorbate for glutamate. In astrocytes, ascorbate transport to the extracellular environment has been linked to glutamate uptake in a process termed “ascorbate-glutamate heteroexchange” that decreases extracellular glutamate levels and, in turn, reduces excitotoxicity and pro-oxidative damage [42]. NMDARs are the essential elements of the mechanism underlying the processes of long-term potentiation (LTP) and long-term depression (LTD), two forms of synaptic plasticity critical for learning and memory [91,92,93]. Physiologically, the protective vitamin C action in the brain is linked with improved neurogenesis, neuronal differentiation and synaptic plasticity [94,95]. Contrarily, vitamin C deficiency has been associated with spatial memory deficits, reduced hippocampal volume and restricted neuronal populations in the dentate gyrus in guinea pigs [96]. Supplementation with vitamin C has been reported to prevent impairments in synaptic plasticity and hippocampal LTP attributable to oxidative damage induced by neurotoxic lead [97].

A plethora of reports concerning the vitamin C/NMDAR relationship strongly suggests that regulation of neurotransmission, as well as potential disturbances in this process, can engage diverse mechanisms that are physiologically linked. Activation of NMDA, AMPA and kainate receptors by extracellular glutamate and concomitant calcium influx can promote the production of ROS. Since neurons are highly sensitive to oxidative damage, the recycling and release of vitamin C appear to be important protective mechanisms during physiological brain activities.

#### 4.1.3. Vitamin C in the Regulation of TRP Channels

The TRP channel superfamily is a heterogenous group of more than 28 representatives that regulate Ca^2+^ influx acting as biosensors and transducers [61]. They are present in the cellular membranes and can be activated by numerous physical and/or chemical signals [98]. Due to their diversity in activation, mechanisms and selectivity, TRP channels can be considered as multiple signal integrators. Among others, TRP stimulation can be triggered by the activation of G-protein-coupled receptors (GPCRs). Moreover, both GPCRs and TRP channels are often co-expressed in the cells. All TRP classical channels are activated through pathways coupled to stimulation of phospholipase C (PLC) [99]. Some TRPs are present in the membranes of intracellular organelles, such as the endoplasmic reticulum, and can induce the additional Ca^2+^ influx through store-operated channels [100,101].

Several lines of evidence suggest that vitamin C has a dual nature and, in addition to antioxidants, it can act as a tissue-damaging pro-oxidant, particularly in cancer cells [102,103]. However, much less is known about the involvement of TRP channels in vitamin C action. Generally, ascorbate in a millimolar concentration in the plasma acts as an oxidant generating hydrogen peroxide (H_2_O_2_), ROS and hydroxyl radicals and, thereby, can negatively affect cell viability [104,105]. In etoposide-resistant and etoposide-sensitive retinoblastoma tumour (RB) cells, which differ by the expression level of several TRP channels, ascorbate induced intracellular Ca^2+^ influx through the activation of pertussis-sensitive G_i/o_-coupled GPCR, which, in turn, activated TRP channels in both cell types [106]. This phenomenon was confirmed by the exposure of RB cells to the pertussis toxin. Moreover, at a 1 mM concentration, when ascorbate acts as a pro-oxidant, larger intracellular Ca^2+^ transients were induced to the etoposide-resistant RB cells with downregulated TRPV1, TRPM2, TRPA1, TRPC5, TRPV4 and TRPM8 channels. Nonetheless, viability in these conditions was compromised in both cell lines.

Antioxidant properties in vitamin C have been observed in HEK-293 cells with a stable expression of human TRPC5 and studied by patch clamp and intracellular Ca^2+^ recording [107]. There are multiple nonspecific stimulators of TRPC5, including receptor agonists (e.g., carbachol and ATP), certain endogenous lipids, redox factors, mild acidification and toxic metal ions. The data showed that vitamin C suppressed only TRPC5 activity that depends on H_2_O_2_, with the lack of effects on other modes of channel activity, confirming the antioxidative mechanism of vitamin C action.

TRPV1 is another calcium-selective channel that is expressed in sensory neurons and mediates noxious stimuli into pain sensation. It can be activated by heat, low pH and a variety of endogenous and exogenous agonists [108]. Increased Ca^2+^ can also activate a negative feedback mechanism by desensitization of TRPV1 by repetitive or sustained stimulation under physiological conditions. The whole-cell patch clamp studies performed on HEK293T cells overexpressing rat TRPV1 and examined under hypoxia (3% of fractional O_2_ concentration) revealed some interesting results regarding vitamin C action [109]. Under hypoxia, the basal TRPV1 current (I_TRPV1_) was partially activated and was increased by vitamin C. However, in the presence of vitamin C applied in batch solution, hypoxia inhibited I_TRPV1_. The authors suggested that the differential response might be caused by the side-specific effects of ROS produced by hypoxia on TRPV1: inhibitory in the extracellular and stimulatory in the intracellular milieu. Vitamin C, when applied to a bath solution, primarily affected the extracellular environment and acted as an antioxidant. With intracellular vitamin C/hypoxia, the stimulatory effects of intracellular ROS may be selectively abolished, leading to the inhibition of I_TRPV1_.

### 4.2. Vitamin C in the Regulation of NO

An interesting relationship between calcium and vitamin C action can be observed during the production and activity of nitric oxide (NO). It is a short-living free radical, lipid-permeable gas synthesized from L-arginine by NOS [110,111]. There are three main isoforms of NOS: neuronal NOS (nNOS), inducible NOS (iNOS) and endothelial NOS (eNOS); they differ by cell and tissue distribution [112]. The enzyme requires several co-factors for full activity, including NADPH, FAD, FMN and BH_4_. All NOS isoforms also bind CaM and contain haem. They can be found in the central as well as peripheral nervous systems with a prevalence of nNOS [113].

In the brain, NO acts as neuromodulator or neurotransmitter in response to different stimuli and controls synaptic plasticity, neurosecretion, learning, memory, neurogenesis and is involved in the processes of LTP and LTD [114,115,116,117]. Triggering of NO production is linked to calcium and is closely associated with the activation of glutamate receptors [118]. Increased intraneuronal Ca^2+^ leads to formation of the Ca^2+^/CaM complex, which binds NOS and initiates NO generation. In this process, a very important role is played by BH_4_ that regulates NOS functions at several levels. BH_4_ increases NOS affinity for substrate, increases haem iron levels and can promote the stability of NOS dimer structure, which is essential for NOS function [119,120,121].

**Figure 2:**
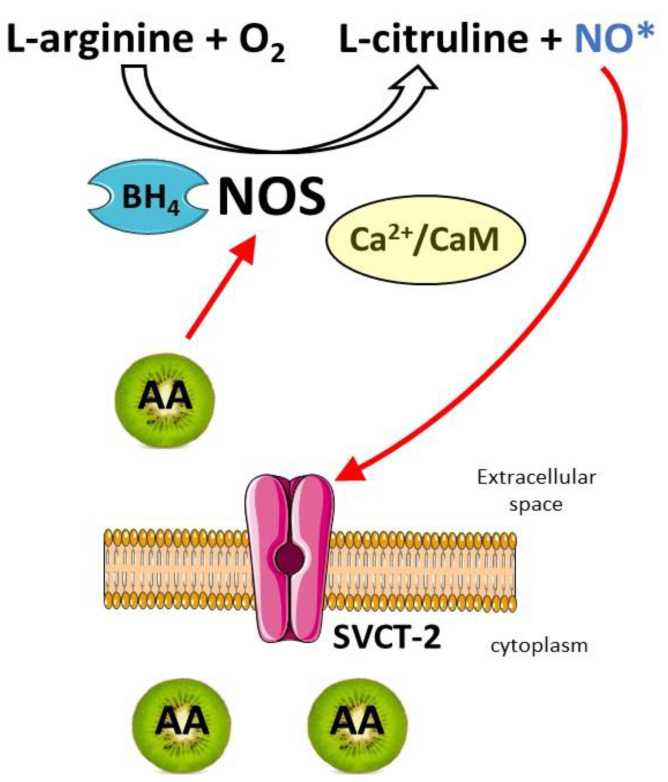
Vitamin C and NO signalling. Increase in Ca^2+^ leads to the formation of Ca^2+^/CaM complex, which binds NOS and stimulates NO production from L-arginine. NO increases the expression of sodium-dependent vitamin C transporter (SVCT-2) allowing for vitamin C intracellular accumulation. Ascorbate also affects regeneration of tetrahydrobiopterin (BH_4_) positively modulating NO generation.

The major physiological target for NO is guanylate cyclase (GC) and low nanomolar NO concentrations stimulate cGMP production [122]. In turn, cGMP activates cGMP-dependent kinases in the cells and, by a subsequent modulation of intracellular Ca^2+^ levels, can regulate many diverse downstream pathways. High level of NO can contribute to excitotoxicity, a critical process in neurodegeneration, which induces oxidative stress and neuronal death. Clarification of Ca^2+^-dependent NMDAR-mediated activation of nNOS allowed us to understand some mechanisms of neurotoxicity observed in many diseases, such as depression, multiple sclerosis, stroke, Alzheimer’s or Parkinson’s diseases [122]. In addition, NOS generates superoxide, which is involved in both cell signalling and injury [123]. High levels of NO can also produce energy depletion, due to inhibition of mitochondrial respiration and inhibition of glycolysis [124]. NO inactivation occurs through its reaction with the superoxide anion (O_2_^−•^) to form the potent oxidant peroxynitrite (ONOO^−^) [125]. This compound can cause oxidative damage, nitration and S-nitrosylation of biomolecules; thus, in the case of L-arginine or BH_4_ deficiency, NOS begins to generate superoxide radicals instead of synthesizing NO [126]. Moreover, as BH_4_ is easily oxidized, an increase in ROS and RNS, such as peroxynitrite, leads to BH_4_ depletion [127]. It should be noted that vitamin C may control NO turnover at its every step. In spite of this, a study on cultured embryonic retinal cells showed an interesting interplay between the ascorbate transport system and NO signalling pathway [49] (Figure 2).

Ascorbate uptake was shown to be tightly controlled by NO and its downstream signalling pathway, but NO also modulated the expression of SVCT-2 and increased the transport capacity for ascorbate. This effect involved the participation of cGMP and PKG. The coupling between NO and ascorbate upon glutamatergic activation was confirmed in the rat hippocampus, indicating the functional relation of both compounds [128]. Under physiological conditions, one of the key functions of vitamin C is the preservation of redox balance and protection from excessive production of ROS by stimulation of glutamate receptors. Vitamin C scavenges ROS produced during normal synaptic activity and neuronal metabolism. Moreover, vitamin C has been shown to accelerate the recycling of BH_4_, which is critical in the synthesis of neurotransmitters, but also for NO generation [129].

A growing body of pre-clinical and clinical data suggests that an increased concentration of extracellular glutamate, with subsequent disturbances in calcium homeostasis, could be linked to depressive-like behaviours, but vitamin C exhibits antidepressant activity [95,130]. The therapeutic effect of ascorbic acid involves modulation of the L-arginine/NO/cGMP signalling pathway [95,131]. Vitamin C can restrict cellular damage due to antioxidant properties but, on the other hand, it may also multiplicate the detrimental effects due to its pro-oxidant potency, leading to the formation of H_2_O_2_ and cell damage. It is worth mentioning that cytotoxicity in vitamin C at high concentrations towards cancer cells can potentially support anti-cancer treatment [132,133].

## 5. Conclusions

The coupling between calcium and vitamin C points to a functional impact from the activities of both compounds in the brain, although several different molecular mechanisms have been identified as well. Two main factors determine vitamin C antioxidative properties: availability of its reduced form—ascorbate and the possibility of dehydroascorbate (oxidized form) re-oxidation. Equally important is a specific mechanism of transport that concentrates vitamin C intracellularly, thereby enhancing its function as the enzyme’s cofactor and antioxidant. Vitamin C has been demonstrated to regulate neurotransmitters, receptors and ion channels, and all these activities are directly or indirectly linked with calcium. The main physiological role of vitamin C is the inhibition of redox imbalance produced by the stimulation of glutamate receptors and a subsequent increase in intracellular Ca^2+^. Interestingly, vitamin C release can be promoted by glutamate in a receptor-dependent manner; thus, glutamate induces an important protective mechanism by regulating the release of this antioxidant. In the brain, glutamatergic activation triggers NO production, but NO is also a modulator of vitamin C release through a heteroexchange mechanism to the extracellular space. Moreover, vitamin C can accelerate the recycling of BH_4_, a co-factor of Ca^2+^/CaM—regulated NOS and several enzymes essential for the synthesis of neurotransmitters. Thus, under physiological conditions, including properly controlled calcium homeostasis, vitamin C should have an active role in the maintenance of neuronal function, actively protecting the brain from abnormal excitability. The apparent complexity of vitamin C mechanisms engaged in neuronal regulation clearly indicates its important role, but a number of questions remain open. Vitamin C does not act solely and coexisting factors and/or compounds, yet undefined, can alter ultimate cell response. Further work examining these potential relationships will greatly enhance our understanding of vitamin C’s role in brain function. Moreover, recognition and precise characterization of neurotoxic molecular events observed in neurodegenerative disorders may facilitate the identification of novel therapeutic targets for vitamin C.
